# Cooperative Suction by Vertical Capillary Array Pump for Controlling Flow Profiles of Microfluidic Sensor Chips

**DOI:** 10.3390/s121014053

**Published:** 2012-10-18

**Authors:** Tsutomu Horiuchi, Katsuyoshi Hayashi, Michiko Seyama, Suzuyo Inoue, Emi Tamechika

**Affiliations:** NTT Microsystem Integration Laboratories, Nippon Telegraph and Telephone Corporation (NTT), Morinosato-Wakamiya, Atsugi-shi, Kanagawa 2430198, Japan; E-Mails: seyama.michiko@lab.ntt.co.jp (M.S.); inoue.suzuyo@lab.ntt.co.jp (S.I.); tamechika.emi@lab.ntt.co.jp (E.T.)

**Keywords:** microfluidics, capillary, flow rate, passive pump

## Abstract

A passive pump consisting of integrated vertical capillaries has been developed for a microfluidic chip as an useful component with an excellent flow volume and flow rate. A fluidic chip built into a passive pump was used by connecting the bottoms of all the capillaries to a top surface consisting of a thin layer channel in the microfluidic chip where the thin layer channel depth was smaller than the capillary radius. As a result the vertical capillaries drew fluid cooperatively rather than independently, thus exerting the maximum suction efficiency at every instance. This meant that a flow rate was realized that exhibited little variation and without any external power or operation. A microfluidic chip built into this passive pump had the ability to achieve a quasi-steady rather than a rapidly decreasing flow rate, which is a universal flow characteristic in an ordinary capillary.

## 1. Introduction

Micro-pumps for micro flow devices are fundamental components and have been studied widely in the field of micro TAS and lab-on-a-chip [1]. This is because it is difficult to reduce the total system size unless a built-in micro-pump is developed even if key devices such as sensors, separators, mixers, and reactors are downsized. An external conventional pump system has many disadvantages in addition to the size, including a large dead volume, a troublesome tube connection, and the need to wash the pump system after every measurement. Microfluidic chips are versatile and their application field can be extended through the use of mechanical micro-pump applied MEMS technology [2–9]. Pumping systems that harness characteristics peculiar to fluids, namely electrophoresis [10–12], electroosmosis [13–15] and electrowetting [16], need electrodes to operate but they have no moving parts.

**Figure 1.**
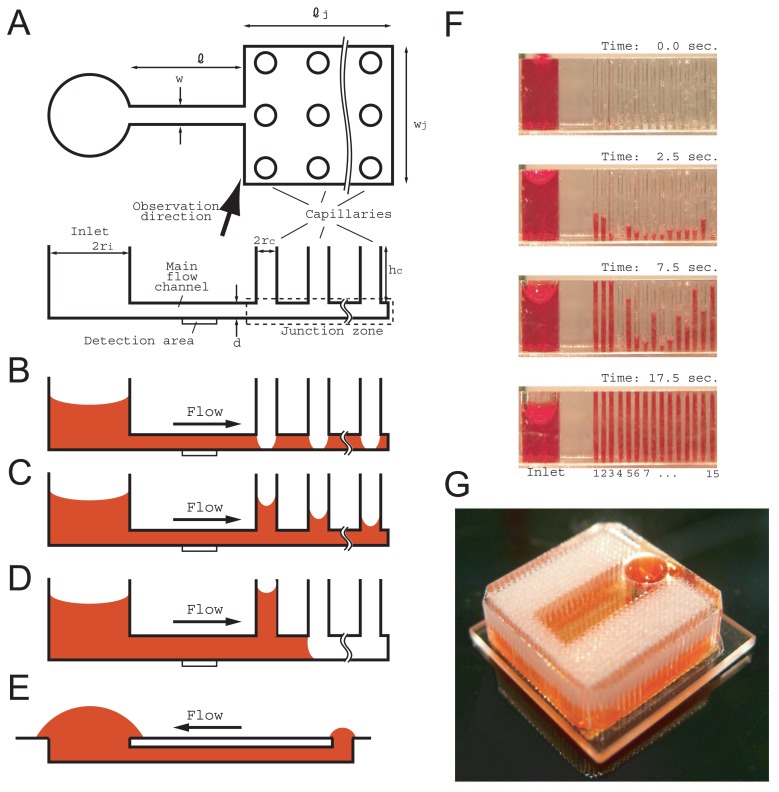
Schematic illustrations of a fluidic chip including a passive pump composed of an integrated vertical capillary array and its suction profile. (**A**) Definitions of position and the notations are summarized in the top and side view illustrations. (**B**) Fluid can propagate in the area of the main flow channel and the junction zone except at the bottoms of the capillaries when *d* < *r_c_*. (**C**) A quasi-steady flow is realized after the B stage. (**D**) No quasi-steady flow is realized if *d* < *r_c_* is not satisfied. (**E**) Comparison with a surface tension pump. (**F**) Time-series imagery of the suction profile of 4-mm-long capillaries in a 3 × 5 arrangement in a square lattice and 2 mm apart; *d*, *r_c_*, and *r*_i_ were 0.05, 0.2, and 1.5 mm, respectively. (**G**) A fluidic chip with a highly integrated vertical capillary array provided a large volume sample for actual sensor application. The main flow channel (not shown) was surrounded by capillaries and an inlet to realize a large flow volume on a small footprint. Sample fluid injected into the inlet propagates in the main channel and branches to the right and left junction zones. The capillaries were arranged in a hexagonal lattice and were 0.35 mm apart. There were 816 capillaries, and the capillary length, *d*, *r_c_*, and *r_i_* were 4, 0.05, 0.14 and 1.5 mm, respectively. Image obtained after several refill operations.

Simple, inexpensive and mass-producible micro-pump systems are required for healthcare, food analysis, and drug discovery where many tests must be performed without cross contamination. A passive pump system, which has no moving parts and no electrodes, is a strong candidate for these application fields where one-time use is preferred. A surface tension passive pump is driven by the pressure difference between two drops of liquid with different radii (Figure 1(E)). The flow rate of this pump is stable and almost constant during long periods of pump operation [17]. Realizing a constant flow rate is effective for improving the performance of micro flow devices and expanding their application fields [18]. The capillary pump is one of the simplest passive pumps, and it has been widely studied for many applications [19–26]. However, a single capillary is limited in terms of flow volume and flow rate. This is because the meniscus advancement rate, *dℓ/dt*, under its own capillary pressure, is inversely proportional to the length, *ℓ*, already filled with liquid [27].

(1) $$\frac{d\ell }{dt}=\frac{\gamma }{\eta }\frac{r}{4\ell }\cos\theta$$

where *r*, *γ*, *η*, and *θ* are the capillary radius, surface tension, viscosity, and contact angle, respectively. The flow resistance is proportional to the length, *ℓ*. The pump structures, where the cavity has many built-in pillars or many branched micro trenches, are fabricated using lithographic techniques [28,29] to control the capillary flow profile. However, the flow volume of these passive pumps is limited because of their two-dimensional structure. In terms of realizing a versatile pump device, a larger flow volume is clearly needed for applications involving complex multiple chemical reactions even if the pump is used in a microfluidic chip. Another desired function for a passive pump is a controllable flow rate.

Passive pumps composed of integrated capillaries, which are formed in the thickness direction of the substrate, have a large flow volume despite their small footprint. We have already developed a single-use microfluidic immunoassay sensor chip that includes a vertical capillary array [30]. Moreover, in terms of flow rate, capillary suction in parallel exhibited a new phenomenon capable of realizing a quasi-steady flow, which we named cooperative suction.

## 2. Experimental

### 2.1. Fluidic Chip Fabrication and Observation of Suction Profile of Vertical Capillary Array

**Figure 2.**
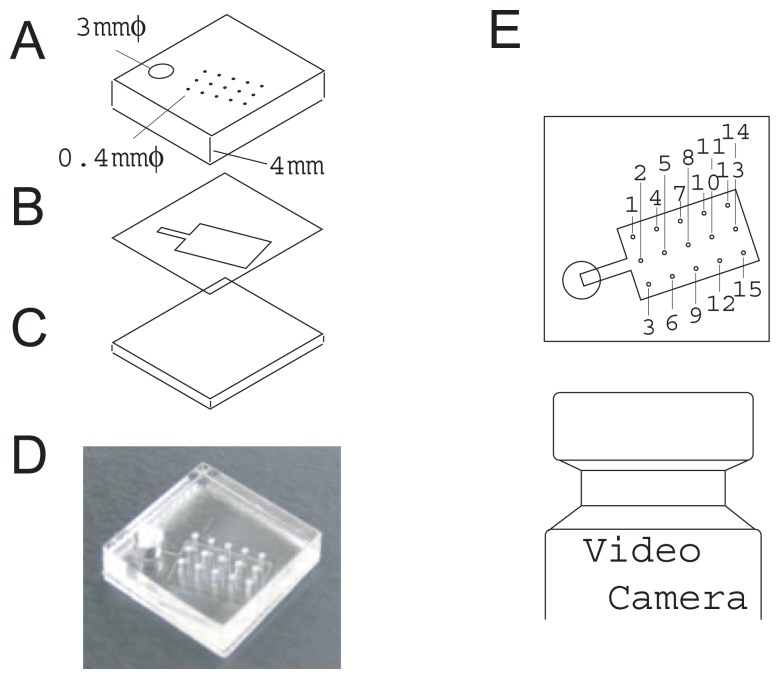
Structure of fluidic chip (A–D) and setup for flow rate measurement (E). An inlet (3 mm diameter) and a vertical capillary (0.4 mm diameter) array were opened in the thick (4 mm) acrylic resin plate (**A**). 15 capillaries were arranged in a square lattice and 2 mm apart. A flow channel was opened in a thin (0.05 mm-thick) two-sided adhesive film (**B**). The bottom of the device was a 1 mm-thick acrylic resin plate (**C**). The assembled flow device is shown in (**D**). Liquid fronts in the inlet and capillaries were observed and recorded from the side of the fluidic chip using a video camera. All the capillaries can be observed without any overlaps from an oblique direction. Individual capillaries are identified by the numbers shown in (**E**). These numbers are common throughout this paper.

All the fluidic chip components were made of polymer consisting of clear acrylic resin (Mitsubishi Rayon Co. Ltd., Japan) and double-sided acrylic resin adhesive films (TL450S-16, 50 *μ*m thickness, Lintec Corporation, Japan). Figure 2(A-C) shows the components of the fluidic chip used for the flow rate experiment. A CO_2_ laser cutting machine (VL 200, Universal Laser Systems Inc., AZ, USA) was used to fabricate passive pump A and bottom plate B from the clear acrylic resin. One side surface of component A was polished flat with sandpaper to solve the problem of capillary image deformation caused by laser cutting. A cutting plotter (CG60ST Mimaki Engineering Co., Ltd., Japan) was used to fabricate the flow channels C of thin two-sided adhesive films to sharpen the cut edge without any protrusion of the adhesive layer. Components A and B were immersed in an aqueous solution of polyalkyleneoxide modified heptamethyltrisiloxane surfactant agent (Silwet L-77, Momentive, Japan) diluted 400 times by volume in a few minutes to obtain uniform wettability. After this treatment, the contact angle of the water on the acrylic resin was reduced from 78 degrees to approximately 65 degrees. These components were dried and fixed together with the two-sided film B. An assembled fluidic chip is shown in (D). The fluidic chip was mounted with its polished side toward a video camera (GZ-HD7-S, Victor, Japan)(E). The fluidic chip was designed so that no capillary overlapped from the polished side view. An aqueous solution of red dye (New Coccine, Kyoritsu Food, Japan) was carefully injected to fill the inlet (28 *μℓ*) with a pipette to minimize the injection shock, and the suction profile of the capillary array was recorded. All the frames of the recorded movie were converted to pictures and liquid front positions were obtained as a function of time using a homegrown image analysis program. A fluidic chip with a highly integrated vertical capillary was fabricated in the same manner but without any polishing.

### 2.2. Simulation

A computational fluidic dynamics (CFD) simulation of a multiphase (air and water) flow using the volume of fluid (VOF) method was carried out with the open source CFD toolbox software, OpenFOAM 1.7.1. [31], to analyze the flow profiles of the fluidic chip. Vertices and surfaces in the base models of the fluidic chips were generated by text based homebrew programs based on an open source computer algebra system, Maxima 5.20.1. [32], in order to generate an optimum mesh efficiently.

The simulations were carried out with models of a smaller fluidic chip than that used in the experiment, because the multiphase flow simulation of the VOF method is extremely time-consuming with our CPU resources, and the small chips allowed us to finish the calculation in less time. Therefore, we use the simulation results to investigate the essential flow profiles rather than to provide an exact fit to the experimental data. The fluidic model used in all the simulations had a virtual hydrophobic capillary on each vertical capillary except for the inlet. This virtual hydrophobic capillary provides overflow protection and simplifies the model. This is equivalent to the contact angle changing discontinuously from the inside of the capillary to the top surface of the fluidic pump. This minor model change did not affect the overall results. The contact angle at each inside wall was set at 60 degrees. A cylindrical water block was installed inside the inlet as an initial condition.

The typical model consisted of 110032 vertices, 82400 cells, and 271701 faces. The simulation was carried out using a workstation (Dell T7400, 2 Xeon CPU (X5492, 3.40 GHz)) or several (4–8) machines (Dell SC1430C, Xeon CPU (E5335 2.00 GHz)) connected by a gigabit network.

## 3. Results and Discussion

### 3.1. Geometrical Parameters

We addressed the issue of flow profile in relation to an incompressible Newtonian fluid, represented by water, in capillaries with the same sub-millimeter order radius. A schematic illustration of the flow path of our target fluidic chip is shown in Figure 1(A). The fluid injected into the inlet propagates in the main flow channel, the junction zone (wide flow channel below capillaries) and the capillaries. The region of interest with regard to the flow rate is located in the main flow channel, represented by the detection area.

The dominant driving force of the flow is the capillary pressure difference between liquid fronts, and the flow rate is determined by the product of the flow resistance between them and the viscosity of the liquid.

Capillary pressures generated in a cylinder capillary (inlet hole, capillaries), *P_cylinder_*, and in a cuboid capillary (main flow channel, junction zone), *P_cuboid_* are expressed as below [27,33].

(2) $$P_{\text{cylinder}}=-2\gamma \cos\theta (1/r)$$

(3) $$P_{\text{cuboid}}=-2\gamma \cos\theta (1/d+1/w)$$

where *r*, *d* and *w* are the radius of the cylinder, and the depth and width of the cuboid, respectively. Here, all the contact angles were set at the same value of *θ* for simplicity. Analytical solutions for the flow resistance of a cylinder channel of length *ℓ, R_cy_*_li_*_nder_* and a cuboid channel of length *ℓ*, *R_cuboid_* are already known [34].

(4) $$R_{\text{cylinder}}=\frac{8\ell }{\pi r^{4}}$$

(5) $$R_{\text{cuboid}}=\frac{4\ell }{w^{2}d^{2}F(d/w)}$$

Here, function *F* is defined,

(6) $$F(s)=\frac{s}{3}-2s^{2}\sum_{n=0}^{\infty }\frac{\text{tanh}\left(\frac{(2n+1)\pi }{2s}\right)}{{\left(\frac{2n+1}{2s}\pi \right)}^{5}}$$

Equations (2–5) are important throughout this paper. In the following discussion, specific forms of the capillary pressure (*P_x_*) and the flow resistance (*R_x_*) are used by setting geometrical parameters, *w*, *d*, *r* and *ℓ*, corresponding to their own shapes.

The flow profile is divided into three stages depending on the location of the fluid front. In the initial stage, the fluid front propagates in the main channel as a result of the capillary pressure difference, *P_m_* − *P_i_*, where *P_m_* is the capillary pressure in the cuboid main flow channel and the *P_i_* is that of the cylindrical inlet. The period of this stage is short and the flow rate is large and decreases rapidly under the control as given by Equation (1). In the second stage, the fluid front propagates in the junction zone. As the fluid front propagates, it changes its convexo-concave shape in a complicated way under the influence of the capillary holes, and fluctuation is expected in the flow rate. However, the absolute value of the capillary pressure in the junction zone |*P_j_*| is estimated as below, because the radii of the convexo-concave shapes are sufficiently greater than the depth of the junction zone for their contributions to be small.

(7) $$\left|P_{m}\right|>\left|P_{j}\right|\approx 2\gamma \cos\theta (1/d)$$

Next, if we limit the capillary pressure, |*P_c_*|, as below, the fluid front is not expected to enter the capillaries until the entire junction zone is filled (Figure 1(B)).

(8) $$\left|P_{j}\right|>\left|P_{c}\right|$$

This is because, even if the fluid enters the capillary, it is pulled back by the larger pressure of |*P_j_*|.

In the last stage, the unoccupied area is the capillaries, and finally all the capillaries are filled to their tops as seen in Figure 1(C). The flow condition from the inlet to the capillaries is expressed as

(9) $$\left|P_{c}\right|>\left|P_{i}\right|$$

The relations between the capillary pressures described above are organized, and we have the following relations between the geometrical parameters needed for a fluid to flow,

(10) $$d<r_{c}<r_{i}$$

where *r_i_*, *r_c_* and d are the inlet and capillary radii and the main channel depth, respectively. The condition *d* < *r_c_* (or Equation (8)) is essential for expressing unique flow rate profiles. If the condition *d* < *r_c_* is not satisfied, the fluid front propagates in both the capillaries and the junction zone concurrently (Figure 1(D)).

**Figure 3.**
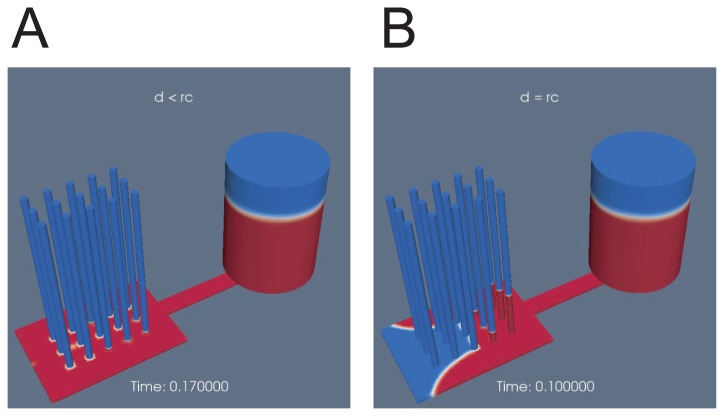
Screen shots of a computational fluidic dynamics simulation. (**A**) Channel depth d is smaller than the capillary radius r (*d* < *r*). Time is 0.17 s. All capillaries waited to draw fluid up until the junction zone was full. (**B**) The channel depth and capillary radius are the same (*d* = *r*). Time is 0.1 s. The fluid front propagated in both capillaries and the junction zone concurrently.

This fluid propagation control realized by controlling the geometrical parameters is confirmed by both experiments and computational fluid dynamics (CFD) simulations of a multiphase (air and water) flow. The simulation (movie S2 left) showed that the water propagated in the junction zone but did not enter the capillaries until 0.17 s. (Figure 3(A)). After 0.17 s, new water fronts generated at the bottom of the capillaries began to appear simultaneously. When *d* < *r_c_* was not satisfied, some capillaries started to draw up before the junction zone filled (Figure 3(B)).

Figure 1(G) shows a fluidic chip including a highly integrated vertical capillary array pump developed for biosensor applications. This fluidic chip satisfied the condition of *d* < *r_c_*. The image was taken 9 minutes after injection. It took about 20 minutes to fill all the capillaries. We confirmed from this image that all the capillaries began to draw fluid once the entire junction zone was filled. None of the capillaries drew fluid to the top but almost all the capillaries were half full.

### 3.2. Cooperative Suction of Capillary Array

**Figure 4.**
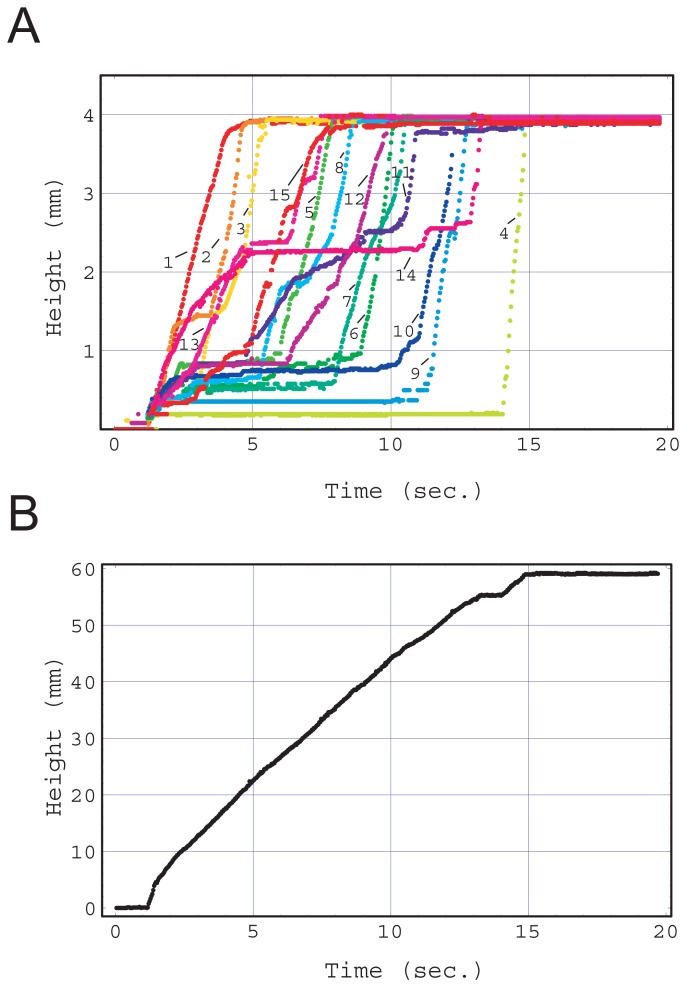
(**A**) Height of fluid front in each capillary obtained by experiment in Figure 1(F) as a function of time after fluid injection. The numbers in the figure are capillary identification labels (See Figure 2(E)). (**B**) Sum of fluid front heights of all capillaries in A as a function of time after fluid injection. The irregular disorganized suction pattern of A was subject to the fact that the increase rate of the sum is constant as shown in B.

Figure 1(F) shows the result of the fluid front rising in each capillary extracted from movie S1. Multiple capillaries drew up fluid simultaneously, however the rising speed differed with regard to time and capillary (See movie S1). The fluid front height in each capillary was obtained as a function of time and is shown in Figure 4(A). It seems that all the fluid fronts rose at different speeds, with different acceleration and timing, and the movements of all the fluid fronts were disordered and independent. However, the sum of all the fluid front heights as a function of time obeyed a simple rule as shown in Figure 4(B). The sum increased in proportion to time after the junction zone was filled, although there were some errors caused by capillary fabrication and image reading. This is equivalent to the flow rate being constant in the main flow channel.

**Figure 5.**
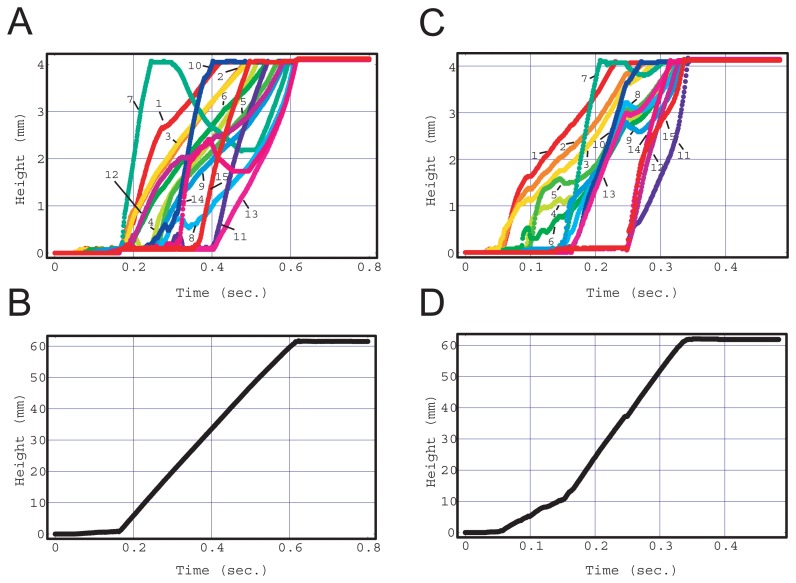
Height of fluid front in each capillary (**A**,**C**) and sum of fluid fronts of all capillaries (**B**,**D**) obtained by CFD simulations as a function of time. The numbers in the figures are capillary identification labels. There are 4-mm-long capillaries in a 3 × 5 arrangement in a square lattice and 0.6 mm apart. *r_c_* and *r_i_* were 0.1 and 1.5 mm, respectively. *d* was 0.075 mm in A and B and 0.1 mm in C and D. The two different slopes in D means that there was no clear separation in the flow profile transition.

**Figure 6.**
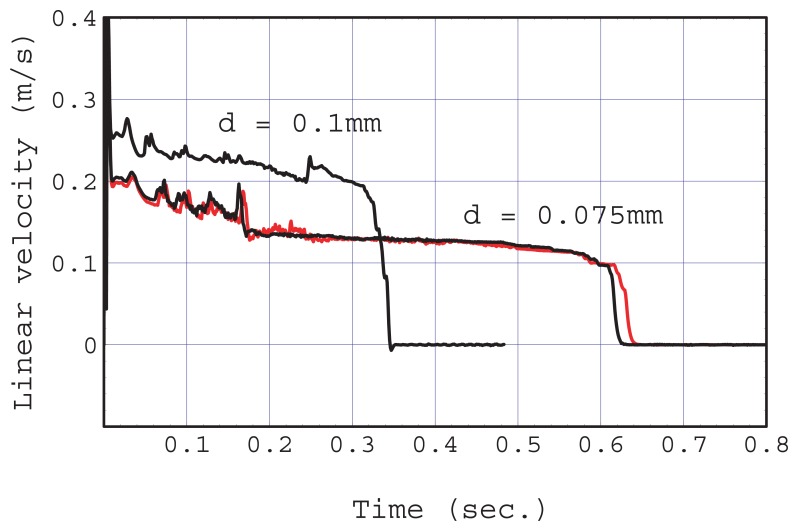
Linear velocity profiles at a height of *d*/2 from the bottom in the region of the detection area obtained by simulations. The flow rate fluctuation was confined to the second stage (before 0.17 s) and after that a quasi-steady flow was observed with the fluidic chip where *d* < *r_c_*, (d = 0.075 mm, black). Simulation conditions were the same except that the gravitational acceleration constant was zero (red). The flow rate fluctuation remained for almost the entire period and the flow rate decreased slightly (d = 0.1 mm).

A simulation provided similar but much clearer results when the condition of *d* < *r_c_* was satisfied as shown in Figure 5(A,B). Figure 6 shows the linear velocity at the cross-sectional center of the main flow channel. The chip that satisfied the condition of *d* < *r_c_* showed a clear transition from the second stage to the third stage at 0.17 s (*d* = 0.075 mm, black). A quasi-steady flow was realized in the third stage, although the velocity fluctuated as a result of the complex motion of the fluid front in the junction zone in the second stage.

In contrast, when the fluidic chip did not satisfy the condition *d* < *r_c_* (*d* = 0.1 mm), the sum of the fluid front heights exhibited two different slopes in Figure 5(D), and there was no clear separation between the second and third stages. The fluctuation in flow velocity remained during the first slope period.

**Figure 7.**
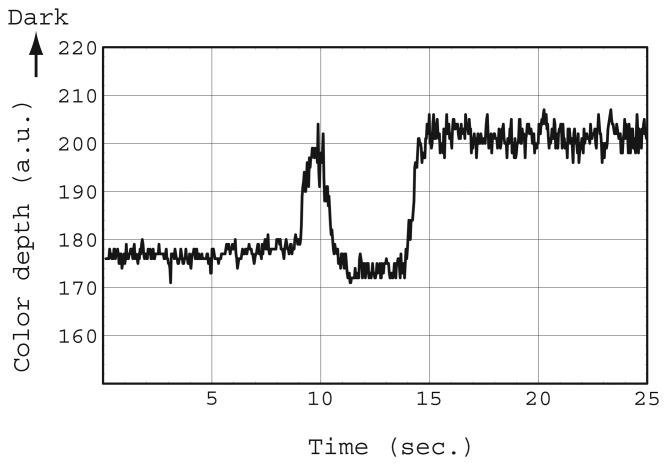
Color depth change of a capillary in the presence of the pullback phenomenon. The color depth corresponds to the liquid front height. A certain capillary exhibited its maximum color depth at 10 s. This value decreased immediately and returned to the value.

In both simulations, some capillaries exhibited a pullback phenomenon. A notable example is capillary #7 in Figure 5(A). This phenomenon was also often observed in the experiment shown in Figure 1(G) (See movie S4). Video was recorded directly above the highly integrated vertical capillary array chip. The center capillary in movie S4 exhibited two reflections of illumination, which means that the liquid front rose to the top of the capillary two times. The color depth change in that capillary as a function of time was obtained using a similar image analysis method to that described in the experimental section (Figure 7). Between the two reflection instances, the color depth was at a low level. This pullback phenomenon is of particular note when d is small, namely there is a large flow resistance from the inlet.

**Figure 8.**
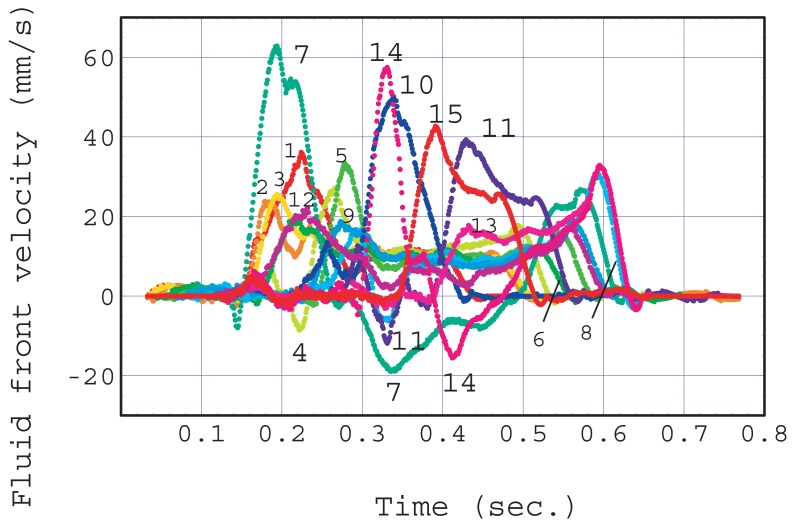
Velocity of fluid front in each capillary calculated using the data in Figure 5(A). A negative velocity indicates the pullback phenomenon. Capillaries #4, 11, 7 and 14 exhibit their maximum negative velocity at 0.22, 0.33, 0.34, and 0.41 s, respectively.

Figure 8 shows the fluid front velocity in each capillary obtained by a numerical derivation of the corresponding fluid front height change shown in Figure 5(A). Pullback phenomena (negative velocity) can be clearly seen in capillaries #4, 11, 7 and 14 and their maximum negative velocities were observed at 0.22, 0.33, 0.34, and 0.41 s, respectively. At the same these times, the capillaries that showed a positive maximum velocity were #7, 14, 10, and both 15 and 11. The combinations of the capillary numbers of these negative maximum and positive maximum velocities, (4, 7), (11, 14), (7, 10), (14, 15 and 11) are the nearest neighbor combinations as shown in Figure 2(E).

The quasi-steady flow velocity is explained based on a qualitative consideration as described below. An individual fluid front in a capillary moves according to the pressure difference, *P_c_* − *P_i_*, and the flow resistance between the fluid fronts of the inlet and that of the capillary. That resistance depends on the position of the capillary in the junction zone and the fluid front heights of the capillary. When the last stage starts, the fluid front height of every capillary is the same. However the distance between individual capillaries and the outlet of the main channel differ. This inequality in flow resistance leads to different rising times. The flow resistance of a capillary depends on the height of the liquid front according to Equation (4). The fluid supply from the main channel is insufficient for all the capillaries. These factors seem to be the origin of the randomness and unpredictability found in the liquid front movement. When the fluid supply from the inlet is limited as a result of the large resistance of the main channel and junction zone, the fluid that had already risen in the capillary is used as the fluid source for the surrounding capillaries because the resistance between nearest neighbor capillaries is small. This is supported by the above discussion of the pullback phenomena. Overall, all the capillaries appeared to cooperate with each other in order to draw the limited fluid supply with maximum efficiency at a certain capillary pressure.

The capillary pressure of each capillary is constant at any given time and is unrelated to the fluid front height, because each capillary radius is the same at any height. Then the average flow rate of the main flow channel at the third stage, *v_av_*, was approximated,

(11) $$v_{av}=\frac{2\gamma \cos\theta }{\text{nwd}R_{\text{eff}}}(\frac{1}{r_{c}}-\frac{1}{r_{i}})$$

Here, the effective resistance, *R*_eff_, is the resistance from the starting point of the main flow channel to the half height of a capillary located at the junction zone center as a representative resistance. This is composed of the flow resistance of cuboid channels (main flow channel, junction zone of half length) and cylindrical channels in parallel (half height capillary array), derived from Equations (5) and (4).

(12) $$R_{\text{eff}}=\frac{4\ell }{w^{2}d^{2}F(d/w)}+\frac{{2\ell }_{j}}{w_{j}^{2}d^{2}F(d/w_{j})}+\frac{1}{n_{c}}\cdot \frac{4h_{c}}{\pi r_{c}^{4}}$$

Here, *ℓ, ℓ*_j_, *w_j_*, *n_c_* and *h_c_* are the main channel length, the junction zone length, the junction zone width, the number of capillaries and the capillary height, respectively. The resistance of the cylindrical inlet is eliminated because its contribution is negligible. Then the total pumping time *T* in the third stage is

(13) $$T=\frac{n_{c}\pi r_{c}^{2}h_{c}}{wdv_{av}}$$

Numerical evaluations of the total pumping time using appropriate values for the geometrical parameters and physical constants were carried out for experimental fluidic chips with 816 capillaries and 15 capillaries and simulation fluidic chips where *d* = 0.075 and *d* = 0.1 mm, and the results were 1330, 9.7, 0.44, and 0.20 s, respectively. These calculated values agreed well with the experiments and simulations.

A simulation result obtained under the same conditions as those used with the *d* = 0.075 mm chip, except that the gravitational acceleration was set at zero, is shown in Figure 6 (*d* = 0.075 mm, red). The flow profile was almost the same as that when the gravitational acceleration was included. The difference in the flow profiles is slightly more visible in stages 1 and 2 than in stage 3. In these stages, the liquid is located in the inlet and the junction zone and is slightly affected by gravitational force because the inlet radius is not very small (*r_i_* = 1.5mm). The pullback phenomenon was also confirmed (movie S3 left). The major driving force of this cooperative suction was confirmed as being the capillary force and not the hydrostatic pressure. A quasi-steady flow is easy to achieve with this pump in a micro-gravity or high-gravity environment.

## 4. Conclusions

We have proposed a novel capillary array passive pump that has sufficient power to overcome weak points in flow profile of single capillary. The pumping principle of our device includes a novel phenomenon, namely a cooperative suction action between capillaries. This was confirmed in an experiment, simulation, and theoretical consideration.

Using our pump as a unit device and integrating a number of these pumps into an array led us to a new concept for designing a flexible flow rate for microfluidic devices. We are currently investigating a flexible flow control method by optimizing the shapes and arrangement of the capillaries.

## Supporting Movies

### Movie S1

Suction profile video of a passive pump consisting of integrated vertical capillaries. Red water is injected into the inlet hole using a pipette at 4 s. The water propagates in the main flow channel and filled junction zone until 6 s. Although it is not shown in the movie, this is indicated by the change in the shape of the water front in the inlet. From 6 s to 20 s the capillary array exhibits an irregular disorganized suction profile, but this can be controlled by a simple rule. The time at which the water is injected is the time origin of Figure 4(A,B).

### Movie S2

Suction profile animation of a passive pump obtained by a computational fluidic dynamic simulation of a multiphase flow using the volume of fluid method. A comparison is made in terms of the difference between the channel depth *d* and capillary radius *r_c_*. The blue, red, and white regions represent air, water and their boundaries, respectively. When *d* < *r_c_*, in the left animation, all the capillaries are waiting to draw fluid until the thin layer beneath them is completely filled. When *d* < *r_c_* is not satisfied, in the right animation, fluid propagates concurrently in the thin layer and the capillaries. Some fluid fronts in the capillaries move up and down in both simulations.

### Movie S3

Suction profile animation of a passive pump obtained by a computational fluidic dynamic simulation of a multiphase flow using the volume of fluid method. The gravitational acceleration is normal (−9.8 m/s ^2^) in the left animation (same as Movie S2 left) and zero in the right animation. Although the suction profile of a capillary array is different, the completion time is almost the same. Some fluid fronts in the capillaries also move up and down in the right animation.

### Movie S4

Pullback phenomenon of liquid front in a vertical capillary observed using the sensor chip shown in Figure 1(G). The video was recorded from directly above. The center capillary shows two reflections of illumination, which means that the liquid front rose twice to the capillary top. The color depth in that capillary reduced at that time. See Figure 7.

## References

[1] Laser D., Santiago J. (2004). A review of micropumps. J. Micromech. Microeng..

[2] van Lintel H., van De Pol F., Bouwstra S. (1988). A piezoelectric micropump based on micromachining of silicon. Sens. Actuators.

[3] Stemme E., Stemme G. (1993). A valveless diffuser/nozzle-based fluid pump. Sens. Actuators A: Phys..

[4] Jeong O.C., Yang S.S. (2000). Fabrication and test of a thermopneumatic micropump with a corrugated p+ diaphragm. Sens. Actuators A: Phys..

[5] Munyan J.W., Fuentes H.V., Draper M., Kelly R.T., Woolley A.T. (2003). Electrically actuated, pressure-driven microfluidic pumps. Lab Chip..

[6] Dong L., Jiang H. (2007). Autonomous microfluidics with stimuli-responsive hydrogels. Soft Matter..

[7] Samel B., Chretien J., Yue R., Griss P., Stemme G. (2007). Wafer-level process for single-use buckling-film microliter-range pumps. J. Microelectromech. Syst..

[8] Blanco-Gomez G., Glidle A., Flendrig L.M., Cooper J.M. (2009). Integration of low-power microfluidic pumps with biosensors within a laboratory-on-a-chip device. Anal. Chem..

[9] Henighan T., Giglio D., Chen A., Vieira G., Sooryakumar R. (2011). Patterned magnetic traps for magnetophoretic assembly and actuation of microrotor pumps. Appl. Phys. Lett..

[10] Aebersold R., Leavitt J., Saavedra R., Hood L., Kent S. (1987). Internal amino acid sequence analysis of proteins separated by one- or two-dimensional gel electrophoresis after in situ protease digestion on nitrocellulose. PNAS.

[11] Lauer H.H., McManigill D. (1986). Capillary zone electrophoresis of proteins in untreated fused silica tubing. Anal. Chem..

[12] Ford S.M., Kar B., Mcwhorter S., Davies J., Soper S.A., Klopf M., Calderon G., Saile V. (1998). Microcapillary electrophoresis devices fabricated using polymeric substrates and X-ray lithography. J. Microcolumn Sep..

[13] Zeng S., Chen C.H., M J.C., Santiago J.G. (2001). Fabrication and characterization of electroosmotic micropumps. Sens. Actuators B: Chem..

[14] Chen C.H., Santiago J. (2002). A planar electroosmotic micropump. J. Microelectromech. Syst..

[15] Debesset S., Hayden C.J., Dalton C., Eijkel J.C.T., Manz A. (2004). An AC electroosmotic micropump for circular chromatographic applications. Lab Chip..

[16] Lee J., Moon H., Fowler J., Schoellhammer T., Kim C.J. (2002). Electrowetting and electrowetting-on-dielectric for microscale liquid handling. Sens. Actuators A: Phys..

[17] Berthier E., Beebe D.J. (2007). Flow rate analysis of a surface tension driven passive micropump. Lab Chip..

[18] Atencia J., Beebe D. (2005). Controlled microfluidic interfaces. Nature.

[19] Juncker D., Schmid H., Drechsler U., Wolf H., Wolf M., Michel B., de Rooij N., Delamarche E. (2002). Autonomous microfluidic capillary system. Anal. Chem..

[20] Chakraborty S. (2005). Dynamics of capillary flow of blood into a microfluidic channel. Lab Chip..

[21] Du W.B., Fang Q., He Q.H., Fang Z.L. (2005). High-throughput nanoliter sample introduction microfluidic chip-based flow injection analysis system with gravity-driven flows. Anal. Chem..

[22] Gervais L., Delamarche E. (2009). Toward one-step point-of-care immunodiagnostics using capillary-driven microfluidics and PDMS substrates. Lab Chip..

[23] Lynn N.S., Dandy D.S. (2009). Passive microfluidic pumping using coupled capillary/evaporation effects. Lab Chip..

[24] Srivastava N., Din C., Judson A., MacDonald N.C., Meinhart C.D. (2010). A unified scaling model for flow through a lattice of microfabricated posts. Lab Chip..

[25] Martinez A.W., Phillips S.T., Butte M.J., Whitesides G.M. (2007). Patterned Paper as a Platform for Inexpensive, Low-Volume, Portable Bioassays. Angew. Chem. Int. Ed..

[26] Miura T., Horiuchi T., Iwasaki Y., Seyama M., Camou S., ichi Takahashi J., Haga T. (2012). Patterned cellulose membrane for surface plasmon resonance measurement. Sens. Actuators B: Chem..

[27] Washburn E.W. (1921). The dynamics of capillary flow. Phys. Rev..

[28] Cesaro-Tadic S., Dernick G., Juncker D., Buurman G., Kropshofer H., Michel B., Fattinger C., Delamarche E. (2004). High-sensitivity miniaturized immunoassays for tumor necrosis factor [small alpha] using microfluidic systems. Lab Chip..

[29] Zimmermann M., Schmid H., Hunziker P., Delamarche E. (2007). Capillary pumps for autonomous capillary systems. Lab Chip..

[30] Horiuchi T., Miura T., Iwasaki Y., Seyama M., Inoue S., Takahashi J.i., Haga T., Tamechika E. (2012). Passive fluidic chip composed of integrated vertical capillary tubes developed for on-Site SPR immunoassay analysis targeting real samples. Sensors.

[31] The open source CFD toolbox. http://www.openfoam.com/.

[32] Maxima, a Computer Algebra System. http://maxima.sourceforge.net/.

[33] Delamarche E., Bernard A., Schmid H., Bietsch A., Michel B., Biebuyck H. (1998). Microfluidic networks for chemical patterning of substrates: design and application to bioassays. J. Am. Chem. Soc..

[34] Carslaw H.S., Jaeger J.C. (1959). Conduction of Heat in Solids.

